# Editorial: Behavioral and experimental health economics

**DOI:** 10.3389/frhs.2022.991135

**Published:** 2022-08-08

**Authors:** Arthur E. Attema, Olivier L'Haridon, Jose Luis Pinto Prades

**Affiliations:** ^1^EsCHER, Erasmus School of Health Policy & Management, Erasmus University, Rotterdam, Netherlands; ^2^Department of Economics, University of Rennes I, Rennes, France; ^3^Department of Economics, University of Navarra, Pamplona, Spain

**Keywords:** behavioral health economics, ambiguity (or knightian uncertainty), time trade off method, altruism, health insurance, experimental economics

Healthcare costs have been increasing worldwide, mainly because of aging and the development of expensive new medical technology. More recently, the COVID-19 pandemic has created an even stronger pressure on healthcare organizations and budgets. Consequently, the efficient allocation of scarce healthcare resources has received high priority. In order to achieve this, correctly measuring and valuing health benefits, with the aid of the relatively new research fields known as behavioral and experimental health economics (1), is pivotal.

Behavioral economics includes the study of risk, ambiguity, time, social and altruistic preferences, and decisions under time pressure. Risk and ambiguity preferences are highly relevant for health economics, because health is surrounded with a lot of uncertainty, both at the diagnostic level and the therapeutic level (2), but also in the context of prevention (3) and health insurance (4, 5). Time preferences are studied because many current decisions have consequences only occurring (far) in the future (6), e.g., vaccinations and other preventive efforts. Social choice is of interest as equity considerations are often given a lot of attention in the health domain, where issues such as equal access to healthcare and the distribution of life expectancy between different socio-economic classes are under scrutiny in many societies (7). Health tends to have a large altruistic component, with many people caring not only about their own health, but also the health of others (8). This is particularly relevant for medical doctors: their degree of altruism can affect the incentive structure that would result in the societal optimal solution (9). The aforementioned preferences are also meaningful because they can largely influence health state valuation methods (10, 11) and they are often related to health-affecting behavior, such as smoking, drinking, and medication adherence (12).

This Research Topic is devoted to some of the newest insights in behavioral and experimental health economics. We collected a diverse set of methodological, experimental, theoretical, empirical, and review studies on health preferences, with a behavioral economic focus. Together, these studies help understanding how expectations, personality traits, self-esteem, ambiguity attitudes, and altruistic preferences shape behavior and care in the health domain.

Liu and Boes investigated the associations between choices of health plans, and traditional individual factors and personality traits. They observe that the latter play a role in predicting health plan choices that is as least as important as common factors such as age, health status, and income. These results support theoretical models integrating insights from behavioral sciences and have implications regarding recent efforts to empower people in improving their health plan choices.

Sevim and Felder  performed a theoretical study that predicts how ambiguity aversion affects the decision to test and treat. Applying the smooth ambiguity model (13) to medical decisions, they show that in case the probability of disease is ambiguous, prior testing becomes more attractive if the default option is no treatment, and less attractive if the default is treatment. Furthermore, they derive that if the probability of a successful treatment is ambiguous, ambiguity aversion reduces the tolerance toward treatment failure, implying that the test option is chosen at a lower probability of failure.

Lipman  reports an experimental study that elicited time tradeoff (TTO) utilities for several health states on behalf of 10-year-old children, as well as the respondents' and 10-year-old children's expected age of dying. The author found that, contrary to previous evidence, there was no relationship between health state values and longevity expectations. Possible explanations for this null result are the psychological distance introduced by the proxy perspective valuation and the methodological differences with previous work.

Aikpitanyi et al.  report a field study investigating the influence of locus of control and self-esteem on the utilization of maternal and child healthcare services in Nigeria, while controlling for other sociodemographic characteristics. They found that women's internal locus of control significantly predicted utilization of antenatal care, skilled birth care, and completion of child vaccination. In addition, having a high self-esteem was a predictor of utilization of antenatal and postnatal care, as well as completion of child vaccination after controlling for other variables. The authors conclude that their results offer important insights for enhancing participants' engagement in intervention programs with the aim to improve maternal and child health outcomes.

Henstock et al. conducted a systematic literature review with the aim to develop an understanding of how behavioral theories have influenced the way preferences for health-related quality of life are elicited and interpreted. Their focus was on the TTO method, being a representation of the quality-adjusted life year concept. They selected three behavioral theories to explore, i.e., expected utility (EU), non-EU, and probabilistic choice theory, while they included order effects as a fourth topic to encompass behavioral theories around timing and sequence of events. The analysis suggests that some ideas transit quickly from economic theory to the TTO literature, e.g., the impact of order effects, but others take longer, such as non-EU models or alternatives to constant discounting. Henstock et al. conclude that researchers within health economics should work more closely with those in mainstream economics and behavioral sciences to accelerate the uptake of new and relevant ideas.

Finally, Neumann-Böhme et al. publish a study on altruism, which analyses the results of a large European-wide COVID survey. The study measured altruism by asking respondents how much they would donate if they would receive a windfall gain equivalent to €1,000. They found that individuals classified as altruistic were more likely to behave pro-socially; e.g., they were more likely to wait at home for test results and to wear facemasks. The authors conclude that adherence to pro-social pandemic behavior may be increased by public health officials emphasizing their altruistic nature.

In sum, this special issue highlights the wide variety of research approaches that are nowadays implemented in the field of health economics. Moreover, the issue covers many of the behavioral economic concepts that are still underexplored in the health domain, including altruism and ambiguity, non-standard preferences (non-EU under risk, violations of constant discounting), and psychological influences. We hope this research collection provides a stepping stone to a wave of new investigations in this challenging discipline.

## Author contributions

AA wrote the first draft of this editorial. OL'H made revisions. All authors approved the final version of the manuscript for publication.

## Conflict of interest

The authors declare that the research was conducted in the absence of any commercial or financial relationships that could be construed as a potential conflict of interest.

## Publisher's note

All claims expressed in this article are solely those of the authors and do not necessarily represent those of their affiliated organizations, or those of the publisher, the editors and the reviewers. Any product that may be evaluated in this article, or claim that may be made by its manufacturer, is not guaranteed or endorsed by the publisher.
